# Second Surgery in Insular Low-Grade Gliomas

**DOI:** 10.1155/2015/497610

**Published:** 2015-10-11

**Authors:** Tamara Ius, Giada Pauletto, Daniela Cesselli, Miriam Isola, Luca Turella, Riccardo Budai, Giovanna DeMaglio, Roberto Eleopra, Luciano Fadiga, Christian Lettieri, Stefano Pizzolitto, Carlo Alberto Beltrami, Miran Skrap

**Affiliations:** ^1^Neurosurgery Unit, Department of Neurosciences, Santa Maria della Misericordia University Hospital, Udine, Italy; ^2^Neurology Unit, Department of Neurosciences, Santa Maria della Misericordia University Hospital, Udine, Italy; ^3^Department of Medical and Biological Sciences, University of Udine, Udine, Italy; ^4^Center for Mind/Brain Sciences (CIMeC), University of Trento, Trento, Italy; ^5^Surgical Pathology Department, Santa Maria della Misericordia University Hospital, Udine, Italy; ^6^Robotics, Brain and Cognitive Sciences Department, Italian Institute of Technology, Genoa, Italy; ^7^Section of Human Physiology, University of Ferrara, Ferrara, Italy

## Abstract

*Background*. Given the technical difficulties, a limited number of works have been published on insular gliomas surgery and risk factors for tumor recurrence (TR) are poorly documented. *Objective*. The aim of the study was to determine TR in adult patients with initial diagnosis of insular Low-Grade Gliomas (LGGs) that subsequently underwent second surgery. *Methods*. A consecutive series of 53 patients with insular LGGs was retrospectively reviewed; 23 patients had two operations for TR. *Results*. At the time of second surgery, almost half of the patients had experienced progression into high-grade gliomas (HGGs). Univariate analysis showed that TR is influenced by the following: extent of resection (EOR) (*P* < 0.002), Δ*VT2T1* value (*P* < 0.001), histological diagnosis of oligodendroglioma (*P* = 0.017), and mutation of IDH1 (*P* = 0.022). The multivariate analysis showed that EOR at first surgery was the independent predictor for TR (*P* < 0.001). *Conclusions*. In patients with insular LGG the EOR at first surgery represents the major predictive factor for TR. At time of TR, more than 50% of cases had progressed in HGG, raising the question of the oncological management after the first surgery.

## 1. Introduction

Due to its challenging technical access [1–9] and until the publication of Yaşargil et al. [9], the insula has been considered surgically inaccessible for a long time. Thanks to a better understanding of the insular functional anatomy, several experiences of insular surgery have been reported in the last decades [3, 5, 7, 8, 10–13]. In addition, recent studies, based on the objective evaluation of the Extent of Resection (EOR), show that this latter is associated with increased overall survival (OS) rates and a delayed tumor progression (PFS) [2, 5, 7, 8, 14–16]. The main limiting factor, in LGGs, the achievement of a radical resection is the involvement of eloquent cortical areas and subcortical functional pathways [17–19].

Although surgery is considered the first therapeutic option [2, 5, 7–9, 11], indications for a second operation in case of TR are still poorly documented [20, 21].

The aim of the study was to determine factors influencing the tumor recurrence (TR) in a cohort of adult patients with an initial diagnosis of insular Low-Grade Gliomas (LGGs) that underwent a second surgery, without any adjuvant treatments between surgeries.

## 2. Methods

### 2.1. Patient Selection

In the present study, we retrospectively reviewed 53 adult patients with insular LGGs, identifying among them a series of 23 cases who underwent a second surgery for TR, between January 2000 and September 2013. To analyse the subpopulation with TR and reduce the selection bias, patients were enrolled on the basis of the following inclusion criteria:

1. Age older than 18 years.
2. LGGs harboring in the insular lobe.
3. Two operations during disease evolution.
4. A period of at least one year between the two operations.
5. Histological confirmation of infiltrative LGG at first surgery.
6. Intraoperative mapping at both first and second surgery.
7. No adjuvant therapy since the first surgery.

The local institution ethics committee on human research approved this study.

### 2.2. Functional Preoperative Assessment and Surgical Technique

**Figure 1:**
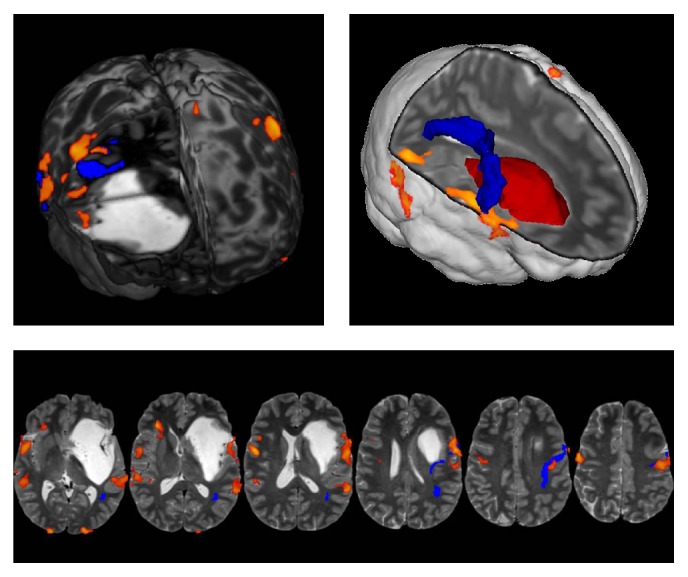
A case of vast left fronto-temporo-insular LGG. Functional MRI/DTI data were overlapped on T2-weighted MRIs and loaded into the Neuro-Navigation system, allowing a better evaluation of the preoperative surgical planning. In the 3D rendering the lesion is represented in red, and the arcuate fasciculus (AF) is in blue, while the orange fMRI spots represent the Broca (anterior spot) and Wernicke area (posterior spot). The functional analysis highlights the cortical areas activated by counting, object naming, and verb generation tasks. The eloquent cortical regions, displaced by the tumor, were easily detected with cortical mapping during surgical procedure. The tumor mass displaces the AF upwards (ACHIEVA 3 T MRI, Philips, Netherlands).

In all cases, high quality 3D T1- and T2-weighted anatomical images as well as functional Magnetic Resonance Imaging (MRI) and diffusion tensor images data were acquired and adopted for the surgical planning and during the surgical procedure itself, after being loaded within a Neuro-Navigation system (Figure 1).

The awake surgery protocol was selected, for both the first and the second surgical procedures, in all cases with lesion harboring on dominant hemisphere, following the methodology previously described by Skrap and colleagues [8].

Moreover, neurophysiological monitoring (MEPs, SEPs, EEG, and ECoG) was employed in all cases following the protocol approved at our institution [14].

### 2.3. Histological and Molecular Analysis

Tumors were histologically reviewed according to the World Health Organization (WHO) classification for tumors of the central nervous system [22]. Immunohistochemistry and FISH analyses were performed on 4 *μ*m thick formalin-fixed paraffin-embedded slides as previously described [23].

Briefly, primary antibodies against Ki-67, GFAP, p53 (Dako), EGFR (Zymed), and IDH1R132H (Dianova) were detected using EnVision FLEX system (Dako). Ki-67 was scored as percentage of positive nuclei. All other markers were qualitatively evaluated as negative or positive. FISH analysis for 1p36 and 19q13 deletions was performed using dual-color 1p36/1q25 and 19q13/19p13 probes (Vysis). IDH gene status and MGMT promoter methylation were assessed on DNA extracted from formalin-fixed paraffin-embedded tissue (QIAamp DNA Mini Kit, Qiagen). IDH1 and IDH2 gene status was evaluated by pyrosequencing as previously reported [23]. After DNA bisulfite conversion with EpiTect Bisulfite Kit (Qiagen), methylation levels of the MGMT promoter in positions 17–39 of exon 1 were investigated, by PyroMark Q96 CpG MGMT (Qiagen) according to the manufacturers' instructions.

### 2.4. Outcome Measures and Follow-Up

After surgery, all patients were clinically evaluated at 1, 3, and 6 months. Subsequently, patients were assessed every six months by both clinical examination and MRI.

The TR has been defined as the demonstration of either unequivocal increase in tumor size or detection of gadolinium enhancement (malignant progression) on follow-up imaging.

Seizure outcome was categorized after first surgical procedure, using the Engel Classification (Class I, seizure-free or only auras since surgery; Class II, rare seizures; Class III, meaningful seizure improvement; and Class IV, no seizure improvement or worsening) [24]. Seizure recurrence in seizure-free patients and seizure worsening in those who continued to experience seizure (i.e., increase in seizure frequency or significant changing in ictal semiology) were considered as a warning sign prompting to a clinical and neuroradiological follow-up.

### 2.5. Volumetric Analysis

All pre- and postoperative tumoral segmentations were performed manually across all MRI slices with the OSIRIX software tool [25] to measure tumor volumes (cm^3^) on the basis of T2 axial slices, as previously described [8, 14]. The extent of glioma removal was evaluated by using MRI images acquired six months after surgery. The EOR was calculated as follows: (preoperative tumor volume − postoperative tumor volume)/preoperative tumor volume [16]. Preoperative Δ*VT2T1* value, a preoperative estimation of the difference between tumor volumes on T2-weighted MRI images and on postcontrast T1-weighted MRI images, was also assessed to define the tumor growing pattern, following the methodological procedure described by Skrap et al. [8].

### 2.6. Statistical Analysis

Characteristics of the study population are described using means ± s.d. or median and range for continuous variables and percentages for categorical variables. Data were tested for normal distribution using the Kolmogorov-Smirnov test. *t*-test or Mann-Whitney test, as appropriate, was used to compare continuous variables. For categorical variables, cross-tabulations were generated and a chi-square or Fisher exact test was used to compare distributions. To describe the time to TR (time between the first and the second surgeries), the Kaplan-Meier approach was used. Patients with not known progression whether malignant or otherwise were censored at the last scan date available. Univariate and Multivariate Cox regression model was used to explore the predictors associated with TR and, consequently, with a second surgery. In Univariate analysis, variables considered as possible predictors of TR were as follows: age, gender, tumor side, preoperative tumor volume, tumor histological subtype, EOR, Δ*VT2T1* value, histological subtype, and molecular markers (Ki-67 (Mib1), GFAP, EGFR, p53, and 1p/19q codeletion; MGMT* promoter methylation*, IDH1-IDH2 mutational status). Considering the small simple size, Multivariate stepwise backward analyses included all variables significant at *P* ≤ 0.05 in Univariate analysis [26]. Results are presented as hazard ratios (HR) and 95% confidence intervals (95% CI). Parametric or nonparametric correlation analyses, as appropriate, were used to explore possible association between TR and seizures after the first surgical procedure. All analyses were conducted with Stata/SE 12.0 for Microsoft Windows. All 2-tailed statistical significance levels were set at *P* < 0.05.

## 3. Results

Baseline demographic, clinical, radiological, and histopathological characteristics of the study population, at the time of first and second surgery, are summarized in Tables 1 and 2, respectively.

### 3.1. Clinical, Radiological, and Histological Data at First Surgical Procedure

The median time between the diagnosis and the first operation was 3.2 months (range 0–11 months). No patient received adjuvant treatment before the first surgical procedure. Preoperative neurological examination was normal in all cases, but all patients were affected by tumor-related epilepsy and required antiepileptic treatment. Before surgery all patients were drug-resistant, according to the ILAE definition [27].

During surgery, when direct electrical stimulation, at subcortical level, did not elicit any functional response, resection continued following the information provided by guided navigation system which remains indicative in subcortical areas. Neuropathological examination resulted in WHO grade II gliomas in all cases. Worsening of the neurological status after surgery was observed in 15 patients. At the six-month follow-up examination, the neurological conditions of all but one patient improved and returned to the initial level. Concerning seizure outcome, 75.5% of patients achieved satisfactory postoperative seizure control (Engel Classes I-II) 6 months after surgery.

### 3.2. Clinical, Radiological, and Pathological Data at the Second Surgical Procedure

A second surgery was performed in 23 patients. The median time between surgeries was 81 months (range 12–144 months). At the time of the second operation, 11 patients, who were seizure-free after the first surgery, had a relapse of unprovoked seizures. Seven patients, who were in Engel class II after the first operation, showed increased seizure frequency and/or ictal semiology worsening. In the remaining 6 cases, tumor relapse was identified on the basis of the MRI follow-up. Postoperative seizure recurrence and worsening were found to be associated with TR (Fisher *P* < 0.001). Considering MRI characteristics, 11 cases showed contrast enhancement, while in 12 cases an increased tumor size was observed through radiological follow-up on T2-weighted images. All tumor recurrences were local.

The preoperative neurological examination was normal in all cases. During surgery, motor function was detected in all cases at both cortical and subcortical level whenever necessary due to the extension of the tumor. No changes in intraoperative MEPs recordings were observed during the whole surgical procedure. Regarding language, we were able to obtain a positive mapping in 85% and 25% of cases at cortical and subcortical level, respectively.

New deficits during the immediate postoperative phase were recorded in 8 cases. At the six-month follow-up examination, the neurological conditions of all but one patient improved and returned to the preoperative level. Histopathological examination showed a progression of the glioma to grade III or IV according to WHO in 17 cases.

Comparison between preoperative MRI enhancement and pathological examination showed that enhancement occurred in 13 out of 17 patients with tumor dedifferentiation. The association between contrast enhancement and the progression to grades III and IV was statistically significant (Fisher *P* = 0.027). Postoperative chemotherapy and radiotherapy were administered in all cases with a diagnosis of glioma grade III or IV.

### 3.3. Volumetric Analysis

**Figure 2:**
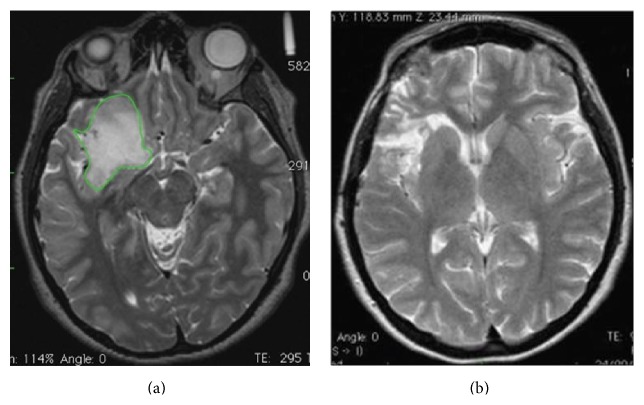
A case of right insular oligoastrocytoma. (a) The preoperative tumor volume computed on postcontrast T2-weighted MRI was 53 cm^3^ (axial slices). The green line represents the area of the tumor before the first surgery. Tumor volume was computed with OSIRIX software. (b) Postoperative tumor residue computed on T2-weighted MRI showed a tumor residual volume of 3 cm^3^ (axial slices). The extent of the tumor volume resection, computed on a T2-weighted MRI sequences, was 94%.

The median preoperative tumor volume at first surgery was 76 cm^3^ (range 5–174 cm^3^) on T2-weighted MRI images, while the median postoperative residual tumor volume, computed on postoperative T2-weighted MRI images, was 12 cm^3^ (range 4–85 cm^3^). Notably, in almost half of the patients at the first surgery, the EOR was higher than 90% (Figure 2). The median extent of tumor volume resection was 83% (range 54–100%).

**Figure 3:**
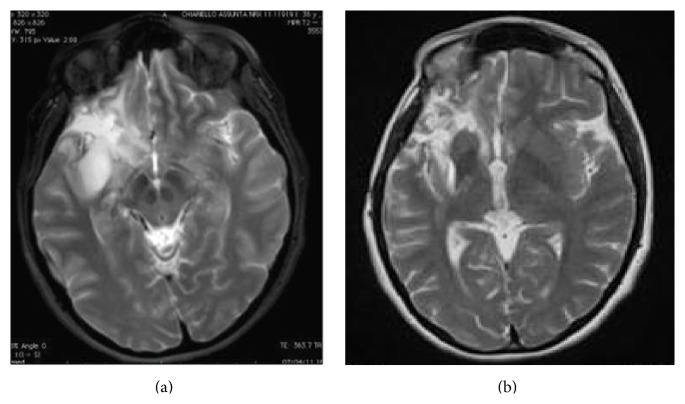
(a) Showing a TR 62 months after the first surgery. The patient did not receive any adjuvant therapy since the first surgery. The volumetric data at first surgery are shown in Figure 2. At second surgery the preoperative tumor volume computed on T2-weighted MRI was 37 cm^3^. Volumetric analysis of postoperative tumor residue computed on T2-weighted MRI showed a tumor residual volume of 4 cm^3^ (axial slices), which has grown mainly in the previous cavity (b). The extent of the tumor volume resection, computed on a T2-weighted MRI sequences, was 91%.

In order to evaluate the role of a diffuse tumor growth pattern on tumor recurrence, preoperative Δ*VT2T1* value was computed in all cases. For this purpose, the study population was divided into two subgroups (subgroup A (37 cases): patients with Δ*VT2T1* value < 30 cm^3^ and subgroup B (15 cases): patients with Δ*VT2T1* value ≥ 30 cm^3^). At second surgery, the median preoperative tumor volume, computed on T2-weighted images, was 40 cm^3^ (range 18–95 cm^3^) (Figure 3). The median extent of tumor volume resection, computed on T2-weighted images, was 82% (range 60–100%). Contrast enhancement, observed before the second operation, was totally removed in all 11 cases.

### 3.4. Risk Factors for Tumor Recurrence

**Figure 4:**
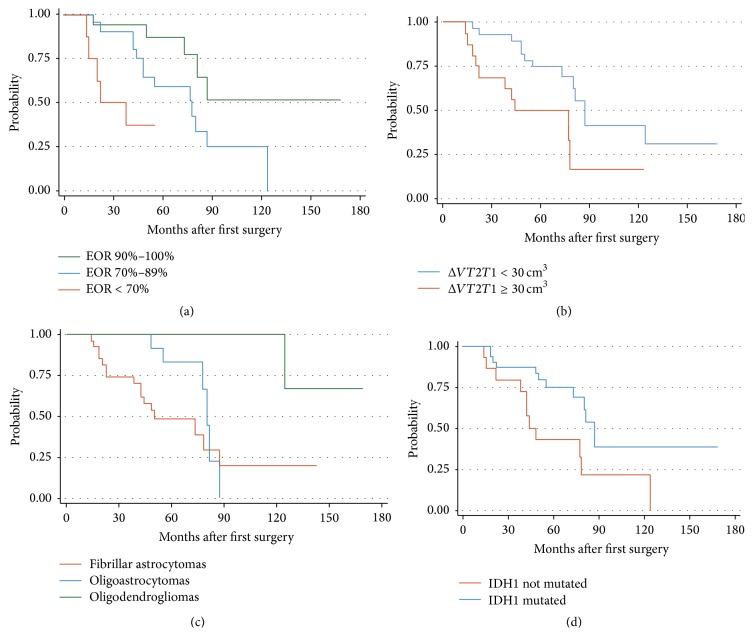
Kaplan-Meier curves revealing TR likelihood in patients with insular Low-Grade Gliomas, stratified by EOR (a), Δ*VT2T1* value (b), histological subtype (c), and IDH1 mutational status (d). Patients with EOR ≥ 90%, Δ*VT2T1* value < 30 cm^3^, diagnosis of oligodendroglioma, and the presence of IDH1 mutation showed significantly lower likelihood of TR after first surgery.

TR was identified in 23 patients. Univariate analysis results are summarized in Table 3. The most important predictor for TR event was the EOR achieved at the first procedure. Patients with TR had a mean EOR of 77.64%; conversely patients without TR had a mean EOR of 90.14% (Mann-Whitney test, *z* + −4.99; *P* < 0.0001). Besides a lower EOR at first surgery (Figure 4(a)), an increase in preoperative Δ*VT2T1* value at the diagnosis (Figure 4(b)), as well as the diagnosis of fibrillary astrocytoma (Figure 4(c)), was associated with higher risk to develop TR. Furthermore, the TR event was decreased for those patients with IDH1 mutation (Figure 4(d)), while the presence of 1p/19q codeletion status was associated with the trend to have a lower risk of TR. In the final model, Cox analysis showed that EOR was the strongest independent predictor for TR (Table 4).

## 4. Discussion

Increasing evidence supports the association between EOR, prolonged OS, and delaying tumor progression [14, 16, 24, 28–40], even for patients with insular LGGs [2, 5, 7–9, 12]. However radical surgery in these cases still remains a critical point, due to insular complex anatomy and functional relationships [1, 2, 8, 18]. Imaging follow-up shows that the residual tumor systematically exhibits a spontaneous and continuous growth, with an inherent risk of anaplastic transformation over the time [18, 41]. While the benefits of an extensive initial resection have been widely demonstrated, the best management of residual tumor still represents an open question [37, 42]. Only two recent investigations analyzed the role of second surgery in case of TR [20, 21] and documented safety and effectiveness of the second surgical procedure in patients with insular LGGs.

### 4.1. Neurological Deficits and Functional Outcome

The major limitation in achieving a radical resection in LGGs surgery is represented by their attitude to infiltrate the subcortical functional pathways [8, 14, 18]. Thus it is a widespread opinion that a second surgery would lead only to an increased risk of new neurological deficits. For the first time Schmidt et al. [21] provided clinical evidence of the safety of a second surgery in 40 patients.

Martino and coworkers analyzed the clinical outcomes of 19 patients with recurrent LGGs in eloquent areas [20], strengthening the concept of possible functional reshaping occurrence after the first surgical procedure [20, 43–45]. In line with these findings, our postoperative neurological results showed that a second surgery is a safe and effective procedure, even for recurrent insular LGGs.

Another possible reason for the positive outcome after a second surgery may be the smaller tumor volume at relapse. This is coherent with the concept of the “*multistage surgical approach*” for LGGs, previously described by Robles et al. [44]. Our investigation also highlights that seizure recurrence in patients who were seizure-free after the first surgery is associated with tumor progression, as previously described by Chang et al. [46].

### 4.2. Surgical Considerations

The overlap of fMRI/DTI data on the T1/T2 3D MRI images in the Neuro-Navigation system is particularly helpful at second surgery, because anatomy with conventional landmarks and functional structures may be significantly modified [8].

Moreover, intraoperative image guidance may also provide critical information during the resection of tumors with a consistency similar to normal brain tissue, by delineating T2-weighted images margins [47]. For this reason, MEPs monitoring was particularly helpful in preventing the direct injury to the posterior limb of the internal capsule and the superior limit towards the corona radiate [48, 49]. Indeed, both structures have been always identified at first and second surgical procedure. On the contrary, subcortical language pathways have been detected in 46% and 25% of cases, at first surgery and second surgery, respectively. Even so, none of our patients developed permanent severe language deficit, supporting the hypothesis of functional reshaping [2, 20, 45, 50, 51]. From a strictly surgical point of view, there are some technical key points to take into consideration at second surgery. At recurrence, there is no endocranic hypertension. Tumor recurrence volume is smaller than the volume at first surgery and the cavity left by the previous operation allows a larger surgical field. The recurrent mass of tumor tissue mainly regrows from the walls of the previous resection into the cavity. We have noticed, also, a better definition between the healthy parenchyma and the tumor tissue, which is softer and, consequently, easier to remove. Moreover, at second surgery, the risk of damaging the vascular structures is much lower, because dissection of the middle cerebral artery (MCA) and its branches has already been performed during the first surgical procedure.

The only difficulty of second surgery is represented by the adhesions. They may cause pain during the opening; moreover, adhesions between* dura mater* and cortex, on the dominant side, may represent a risk of damage to the cortical language areas. In conclusion, the newly infiltrated deep tumoral tissue is not resected if it has been shown to still be functional based on brain mapping results.

### 4.3. Risk Factors for Tumor Progression

The idea of performing a new procedure during regrowth of LGGs, before anaplastic transformation, has been proposed in order to obtain a greater impact on survival [21, 32, 44, 52]. Thus, we tried to identify factors that could provide an early identification of those patients with higher possibility to develop TR. Our findings indicated that the time to TR, even among insular LGGs, is longer in patients who underwent wider resections, as previously demonstrated.

These results support the idea that tumors with larger residual postoperative volume may have an inherently faster growth; therefore, they may recur earlier in the setting of a subtotal resection [15, 16]. Moreover this investigation confirmed the data we previously reported about the role of Δ*VT2T1* value on TR: patients with preoperative Δ*VT2T1* value more than 30 cm^3^ have an earlier TR (*P* value = 0.001). In fact this value reflects a lower possibility to obtain a higher EOR.

McGirt et al. showed that patients with oligodendroglioma and oligoastrocytoma have a better prognosis compared to those with fibrillary astrocytoma [32]. To support this result we separated these histological subtypes confirming that oligodendrogliomas have more benign courses [8, 53, 54].

Regarding the molecular analysis, recent data demonstrated that LGGs display a variety of molecular alterations that may have predictive or prognostic value [55, 56]. The molecular data pointed out that the risk of TR was significantly reduced in the presence of IDH1 mutation, as previously demonstrated by Gozé et al. [56, 57], while the presence of 1p/19q codeletion status was associated with a lower TR risk trend. The prognostic value of IDH1/IDH2 mutations is more controversial. Otherwise 1p/19q codeletion status in LGGs has been widely demonstrated to be associated with a favorable outcome whatever the endpoint: overall survival, progression-free survival, or spontaneous tumor growth velocity [37, 53, 56, 58]. Furthermore, the molecular analysis evidenced that Ki-67 value, as well as p53, GFAP, EGFR, and MGMT status, does not influence the risk for TR after the first surgical procedure, suggesting that other molecular markers should be selected for early identification of patients with a major risk of TR [23]. In closing, the Multivariate analysis highlighted that the only independent factor associated with TR is represented by EOR at first surgery, confirming the findings reported in the literature [5, 8, 14, 24].

As far as the methodological procedure is concerned, the present investigation has potential limitations. First, it is a retrospective study; thus it is limited in nature. Patients with recurrence insular LGGs that are suitable for second surgery are* per se* highly selected. Thus, the number of our samples is limited, but, if we consider the papers, mentioning insular second surgery [2, 5, 7, 12], the overall number of patients is 32; thus our study population in a singular institution (23 patients) is not considerably small and it is statistically sufficient to draw some preliminary considerations, which need to be confirmed by enlarging the case study. Moreover, insular surgery is rare at first diagnosis and even rarer at second surgery, so it is not easy to find large population in literature. In any case, it is unlikely that a prospective, randomized study will be designed to address these issues; thus, we believe retrospective, matched studies or prospective observational trials may be a more practical solution, as previously described [15]. Our findings should be validated in a wider series, using multi-institutional cohort to create a potential model able to stratify the risk of TR after the first surgery. In this way, it would be possible to anticipate adjuvant postoperative treatments, also in patients with a diagnosis of pure LGG.

The timing of second surgery has not been well defined yet. Anyway, as previously remarked by Martino et al, it is better to “overindicate” an early second surgery than performing a late surgery when the tumor has already transformed into high-grade gliomas, especially in consideration of the low morbidity profile associated with reoperation [20].

## 5. Conclusions

In insular LGGs patients, the EOR at first surgery represents the major predictive factor for TR. Further molecular analysis will be necessary to better stratify patients in terms of risk for TR, thus identifying patients that could benefit from an early adjuvant treatment after the first surgical procedure.

## Figures and Tables

**Table 1:** Demographic, clinical, neuroradiological, and pathological data at first surgery.

Parameter	Value
Number of patients	53
Sex	
Female	23 (43.40%)
Male	30 (56.60%)
Mean age (yrs)	38 (range 19–69)
Tumor side	
Left	36 (67.92%)
Right	17 (32.08%)
Median preoperative T2 tumoral volume in cm^3^ (range)	76.33 (range 5–174)
Median preoperative Δ*VT2T1 value* in cm^3^ (range)	23.13 (range 1–112)
Δ*VT2T1* category	
<30 cm^3^	37 (69.81%)
≥30 cm^3^	16 (30.19%)
Intraoperative protocol	
Awake surgery	41 (77.36%)
General anesthesia	12 (22.64%)
Cortical mapping	
Speech arrest and motor function orbicularis oris	All 41 cases with lesion involving the dominant hemisphere
Slurred speech or dysarthria	26 (49%)
Anomia	26 (49%)
Subcortical mapping	
Identification of corticospinal tract as posterior edge of resection	All cases
Identification of subcortical language pathways	Positive sites were detected in 24 cases (45.3%)
Neurophysiological data	
Reversible reduction of MEPs amplitude	7 out of 10 patients, who developed postoperative transient motor deficit
Irreversible MEPs loss	In 1 patient who showed, after surgery, a permanent motor deficit
Median EOR in % (range)	82.98 (range 54–100)
EOR category	
≥90%	22 (41.51%)
70–89%	23 (43.40%)
<70%	8 (15.09%)
Immediate postoperative clinical findings	
No deficits	37 (69.81%)
Neurological deficits	15 (30.19%)
Motor deficits	9 (16.98%)
Speech disorders	6 (13.21%)
Clinical outcome 6 months after surgery	
No deficits	52 (98.11%)
Neurological deficits	1 (1.89%)
Postoperative Engel Class 6 months after surgery	
I	36 (67.92%)
II	4 (7.55%)
III	8 (15.10%)
IV	5 (9.43%)
Histological diagnosis	
Fibrillary astrocytoma	31 (58.5%)
Oligodendroglioma	6 (11.3%)
Oligoastrocytoma	16 (30.2%)
Molecular profile	
Mib1-Ki-67 expression	3.5% (range 1–5%)
1p/19q codeletion presence	13 (25%)
P53 expression	33 (62.26%)
IDH1 mutation	45 (85%)
MGMT promoter methylation	39 (73.58%)

**Table 2:** Summary of characteristics at tumor recurrence in the subgroup of patients who underwent a second surgery.

Parameter	Value
Number of patients	23
Sex	
Female	8 (34.78%)
Male	15 (65.22%)
Mean age (yrs)	42 (range 25–54)
Tumor side	
Left	15 (65.22%)
Right	8 (34.78%)
Median time to tumor recurrence	81 months (14–124)
Seizures relapse at second surgery	11 (47.83%)
New contrast enhancement before second surgery	11 (47.83%)
Intraoperative protocol	
Awake surgery	16 (69.57%)
General anesthesia	7 (30.43%)
Immediate postoperative findings	
No deficits	15 (65.22%)
Neurological deficits	08 (34.78%)
Motor deficits	4 (17.39%)
Speech disorders	3 (13.04%)
Visual field disorders	1 (4.35%)
Clinical outcome 6 months after surgery	
No deficits	22 (95.65%)
Neurological deficits	1 (4.35%)
Histological data	
LGGs (WHO II)	6 (26.09%)
Anaplastic gliomas (WHO III)	7 (30.43%)
Glioblastomas (WHO IV)	10 (43.48%)
Mib1-Ki-67 expression	16.5% (range 2–70%)

**Table 3:** Univariate analysis of clinical and volumetric tumor data and histological and molecular parameters with tumor recurrence in patients with insular LGGs.

Factor	Tumor recurrence		
	HR	95% CI	*P*
Age (modelled as continuous variable)	1.009	0.973–1.047	0.608
Sex			
Male	1		
Female	1.368	0.595–3.415	0.460
Tumor site			
Left	1		
Right	0.886	0.359–2.183	0.792
Preoperative T2 tumor volume cm^3^ (modelled as continuous variable)	1.005	0.994–1.016	0.399
Tumor subtype			
Fibrillary astrocytoma	1		
Oligoastrocytoma	0.509	0.195–1.324	0.166
Oligodendroglioma	0.081	0.011–0.643	**0.017**
% EOR (modelled as continuous variable)	0.932	0.899–0.965	**0.000**
% EOR			
≥90	1		
70–89	2.792	0.938–7.931	**0.052**
≤69	8.936	2.302–34.687	**0.002**
Δ*VT2T1* (modelled as continuous variable)	1.045	1.022–1.068	**0.000**
Δ*VT2T1*			
<30 cm^3^	1		
≥30 cm^3^	2.950	1.243–7.001	**0.014**
Mib1-Ki-67 (modelled as continuous variable)	0.931	0.722–1.201	0.585
1p/19q codeletion *Presence versus absence*	0.288	0.082–1.003	**0.051**
P53 mutation (modelled as continuous variable)	1.006	0.994–1.018	**0.317**
EGFR (modelled as continuous variable)	0.995	0.983–1.008	0.946
GFAP (modelled as continuous variable)	1.007	0.993–1.021	0.323
IDH1 *Mutation versus no mutation*	0.383	0.168–0.872	**0.022**
MGMT *Promoter methylation versus no promoter methylation*	0.912	0.375–2.215	0.840

HR, hazard ratio; CI, confidence interval; EOR, extent of surgical resection; Δ*VT2T1*, volumetric difference between preoperative tumor volumes on T2- and T1-weighted MRI images.

Boldfacing represents statistical significance values (*P* < 0.05) obtained from two-sided tests (Cox regression).

**Table 4:** Variable independently associated with tumor recurrence after surgical resection of insular LGGs in a multivariate proportional hazards analysis (Cox model). In the final model, the EOR remained the strongest independent significant predictor of TR after first surgery for insular LGG.

Factor	Tumor progression		
	HR	95% CI	*P*
% EOR (modelled as continuous variable)	0.930	0.895–0.967	**0.001**

EOR = extent of surgical resection.

## Abbreviations

DES: Direct electrical stimulation
ECoG: Electrocorticography
EEG: Electroencephalography
EOR: Extent of Resection
DICOM: Digital Imaging and Communications in Medicine (standard)
DTI: Diffusion tensor imaging
fMRI: Functional MRI
KPS: Karnofsky Performance Scale
IES: Intraoperative electrical stimulation
LGGs: Low-Grade Gliomas
MEPs: Motor evoked potentials
MRI: Magnetic Resonance Imaging
SEP: Somatosensory evoked potentials
TR: Tumor recurrence
Δ*VT2T1*: Volumetric difference between preoperative tumor volumes on T2- and T1-weighted MRI images.
